# CRISPR/Cas9-Mediated Rapid Generation of Multiple Mouse Lines Identified *Ccdc63* as Essential for Spermiogenesis

**DOI:** 10.3390/ijms161024732

**Published:** 2015-10-16

**Authors:** Samantha A. M. Young, Haruhiko Miyata, Yuhkoh Satouh, Hirotaka Kato, Kaori Nozawa, Ayako Isotani, R. John Aitken, Mark A. Baker, Masahito Ikawa

**Affiliations:** 1 School of Environmental and Life Science, University of Newcastle, Callaghan, New South Wales 2308, Australia; E-Mails: samantha.a.young@uon.edu.au (S.A.M.Y.); john.aitken@newcastle.edu.au (R.J.A.); mark.baker@newcastle.edu.au (M.A.B.); 2 Research Institute for Microbial Diseases, Osaka University, Suita, Osaka 565-0871, Japan; E-Mails: hmiya003@biken.osaka-u.ac.jp (H.M.); yuhkohs@biken.osaka-u.ac.jp (Y.S.); hkato@biken.osaka-u.ac.jp (H.K.); nozawak@biken.osaka-u.ac.jp (K.N.); 3 Graduate School of Medicine, Osaka University, Suita, Osaka 565-0871, Japan; 4 Immunology Frontier Research Center, Osaka University, Suita, Osaka 565-0871, Japan; E-Mail: isotani@biken.osaka-u.ac.jp

**Keywords:** genome editing, targeted mutagenesis, spermatogenesis, sperm motility

## Abstract

Spermatozoa are flagellated cells whose role in fertilization is dependent on their ability to move towards an oocyte. The structure of the sperm flagella is highly conserved across species, and much of what is known about this structure is derived from studies utilizing animal models. One group of proteins essential for the movement of the flagella are the dyneins. Using the advanced technology of CRISPR/Cas9 we have targeted three dynein group members; *Dnaic1*, *Wdr63* and *Ccdc63* in mice. All three of these genes are expressed strongly in the testis. We generated mice with amino acid substitutions in *Dnaic1* to analyze two specific phosphorylation events at S124 and S127, and generated simple knockouts of *Wdr63* and *Ccdc63*. We found that the targeted phosphorylation sites in *Dnaic1* were not essential for male fertility. Similarly, *Wdr63* was not essential for male fertility; however, *Ccdc63* removal resulted in sterile male mice due to shortened flagella. This study demonstrates the versatility of the CRISPR/Cas9 system to generate animal models of a highly complex system by introducing point mutations and simple knockouts in a fast and efficient manner.

## 1. Introduction

Spermatozoa are highly specialized cells whose role is to deliver genetic information to an oocyte. Following ejaculation the sperm flagellum moves in a well characterized and organized manner [1], resulting in the progressive movement of the cell towards the oocyte. Sperm flagella are similar in structure to the cilia of somatic cells, and are comprised of the axoneme made up of two central singlet microtubules surrounded by a ring of nine doublet microtubules, known as the 9 + 2 structure [2,3]. The movement of the flagella occurs by active sliding of doublet microtubules by a group of proteins called axonemal dyneins [2]. The axonemal dyneins are classified into the inner arm dyneins (IAD) and the outer arm dyneins (OAD) depending on where on the doublet microtubule they are located. Both IADs and OADs are composed of several heavy, intermediate and light chain subunits [4,5].

The components and functions of these dyneins are well characterized in the unicellular, biflagellate algae *Chlamydomonas reinhardtii* [6]. The dynein components of this organism are highly conserved between this and most mammalian species including mice and humans (Table S1), suggesting their importance in regulating flagellar activity. However, *Chlamydomonas* is not an ideal organism to study in terms of understanding mammalian spermatozoa due to the presence of the double flagella, and the asymmetric beat pattern, which more closely resembles that of cilia. Furthermore, electron micrographs demonstrate that mammals have additional flagellar structures, with the axoneme surrounded by outer dense fibers (ODFs), a mitochondrial sheath in the midpiece and a fibrous sheath in the principal piece, all of which is not present in more primitive species [2].

Diseases involving dynein motor proteins in humans are often highly complex. Some patients with the genetic disease Primary Ciliary Dyskinesia (PCD) present with male infertility, due to problems in the formation or function of these dynein complexes [5,7,8,9,10,11]. However, not all patients with PCD have fertility issues [12]. This could be due to the complexity of the disease as well as the different expression profiles of the dynein related genes. Thus, there is a dearth of information in regards to the regulation of dynein motor units as well as the function of a majority of dynein genes in mammals.

The advent of gene manipulation systems such as the CRISPR/Cas9 (clustered regularly spaced palindromic repeats (CRISPR) and the CRISPR associated proteins (Cas)) technology now allows us to investigate not only the role of individual gene products but to also determine the role of individual regions of the gene in a short span of time. CRISPR mediated non-homologous end joining (NHEJ) can generate simple knockouts (sKO) and co-injection with an oligonucleotide containing the desired mutation can generate mice with this mutation via homology directed repair (HDR) in an efficient manner [13,14,15]. In this study, we manipulated three dynein complex genes (*Dnaic1*, *Wdr63* and *Ccdc63*) to investigate their functions in mice using variations of the CRISPR/Cas9 system. These genes were selected based on location within the dynein complex, their strong expression in the testis, phosphorylation state and/or their connection to the human disease, PCD.

Dynein axonemal intermediate chain 1 (*Dnaic1*) is the homologue of *Chlamydomonas* intermediate chain 78 (*IC78*) that is a component of the OAD [5]. A sKO of *Dnaic1* has already been achieved [16,17], however due to the lethality of this sKO within 5–10 days of birth, conditional KO (cKO) mice were generated that had *Dnaic1* deleted upon treatment with tamoxifen [18]. These cKO mice did present with a phenotype similar to PCD [18] however the fertility of these mice was not examined. DNAIC1 was found to be phosphorylated at the point in epididymal maturation where spermatozoa acquire motility; specifically the phosphorylation of serine 124 and 127 in rats [19,20]. The same residues have been reported to be phosphorylated in mice [21]. Another intermediate chain dynein of OAD is DNAIC2 (IC69 in *Chlamydomonas*). This protein is not reported to be phosphorylated in mouse spermatozoa. A double amino acid substitution of the phosphorylated residues (S124A, S127A) in *Dnaic1* was undertaken to investigate the function of these two residues in male fertility utilizing the HDR function of the CRISPR/Cas9 system.

WD repeat domain-containing 63 (*Wdr63*) is the homologue of *Chlamydomonas* intermediate chain 140 (*IC140*) that is a component of the IAD [22]. The IAD are thought to be responsible for bend formation and propagation in the flagella [23]. *Wdr63* is also conserved and named *IC116* in the ascidian *Ciona intestinalis*. In *Ciona*, sperm activation and chemotaxis to the egg are induced by an egg-derived substance called SAAF (sperm-activating and attracting factor) [24,25]. IC116 is dephosphorylated when *Ciona* spermatozoa are activated by SAAF, suggesting the importance of IC116 to regulate sperm motility [24]. As this gene has not been knocked out in the mouse we utilized CRISPR/Cas9 with two sgRNAs to target a region of the *Wdr63* locus spanning two exons, resulting in a deletion of the flanked region.

Coiled-coil domain-containing 63 (*Ccdc63*) and 114 (*Ccdc114*) are the homologues of *Chlamydomonas DC2* that is part of the outer dynein arm-docking complex (ODA-DC) [9]. In *Chlamydomonas*, ODA-DC was shown to be involved in the periodic OAD arrangement at specific positions on the microtubules [26]. *CCDC114* has been implicated in several cases of PCD in humans, and it has been shown that loss of *CCDC114* results in a lack of OADs in human respiratory epithelial cells [9]. A study investigating the loss of *CCDC114* showed patients presenting with PCD but no fertility loss, potentially due to compensation by *CCDC63* that is strongly expressed in the testis [9]. As with *Wdr63* no KO mouse model exists for this gene, therefore the NHEJ function from a single sgRNA for CRISPRS/Cas9 was utilized to generate sKO mice.

By combining several uses of the CRISPR/Cas9 system, generating a specific mutation (*Dnaic1*) or creating an sKO (*Wdr63*; two sgRNAs and *Ccdc63*; single sgRNA) we have demonstrated its versatility and effectiveness in analyzing several components of a complex system, saving time and increasing understanding of this essential group of proteins for male reproduction.

## 2. Results and Discussion

### 2.1. Localization of Target Genes

**Figure 1:**
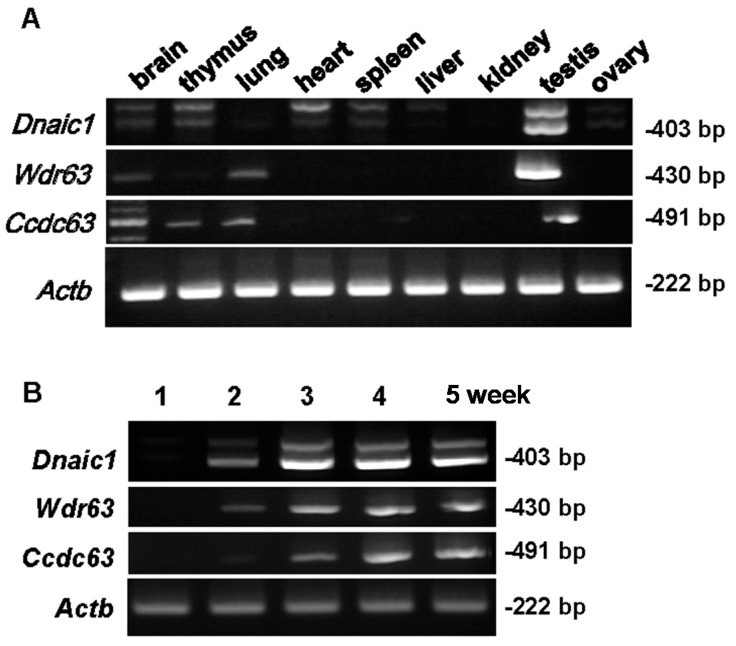
RT-PCR for *Dnaic1*, *Wdr63*, and *Ccdc63* primer sets. (**A**) cDNA library for various tissues; (**B**) cDNA from mouse testes at 1–5 weeks postnatal; *Actb* (β*-actin*) as control. Size of band shown on the right.

RT-PCR was conducted to analyze the expression of our target genes. All three genes were strongly expressed in the testes (Figure 1A). Weak *Dnaic1* expression could also be seen in brain, thymus, heart and spleen tissues (Figure 1A). It should be noted that the extra (upper) band visible for *Dnaic1* is not of the correct size and is most likely a non-specific band or splicing variant that has not been reported in the literature. *Wdr63* could be detected in brain and lung tissues, while *Ccdc63* could be detected in brain, thymus and lung, albeit to a lesser degree (Figure 1A).

We further performed PCR for each gene using testicular cDNAs obtained from 1-, 2-, 3-, 4- and 5-week-old mice (Figure 1B). *Dnaic1* and *Wdr63* are first apparent at 2 weeks, with expression increasing at 3 weeks for *Dnaic1*. In contrast, *Ccdc63* is first seen at three weeks, indicating that *Ccdc63* is incorporated into the dynein complex later in spermatogenesis.

### 2.2. Analysis of Dnaic1 Double Point Mutation Phenotype

#### 2.2.1. Generation of *Dnaic1* Mutant Mice

**Figure 2:**
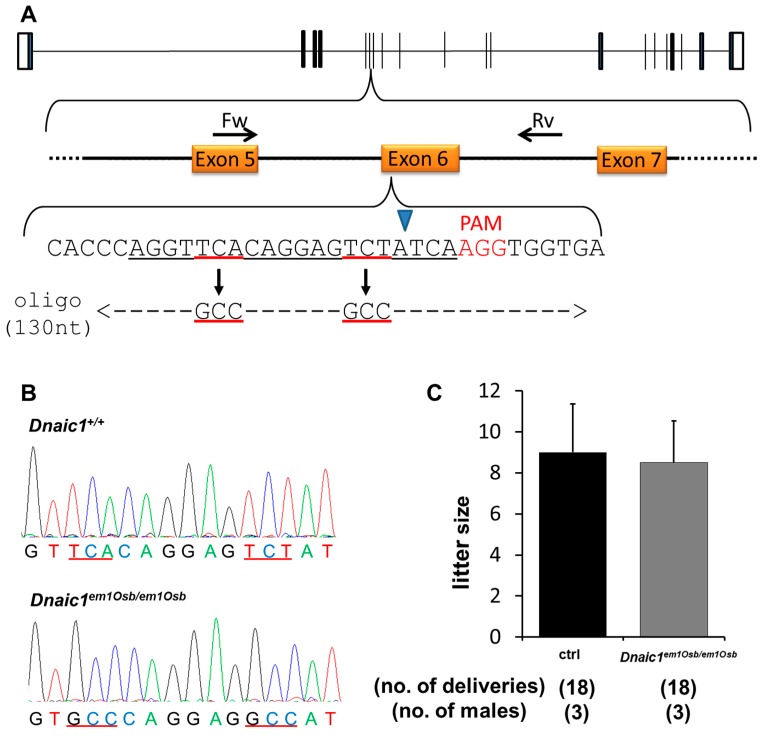
CRISPR/Cas9 strategy and results for *Dnaic1^em1Osb/em1Osb^* male mice. (**A**) *Dnaic1* exon 6 was targeted; coding exons shown as solid black bars, non-coding exons shown as open bars. Forward (Fw) and reverse (Rv) primers for validation and genotyping shown; black arrows labelled Fw and Rv. sgRNA sequence; underlined in black. Mutation targets underlined in red. PAM sequence is shown in red. Blue arrowhead indicates site of Cas9 digestion. Single strand oligonucleotide containing desired mutation; underlined in red, dashed line indicates sequence homologous to wild-type *Dnaic1* sequence; (**B**) Sequencing of *Dnaic1^em1Osb/em1Osb^* mice; targeted mutation underlined in red; (**C**) Natural mating analysis of *Dnaic1^em1Osb/em1Osb^* male mice with WT females.

To achieve a double amino acid substitution of serine 124 and serine 127 to alanines (S124A and S127A) in the *Dnaic1* gene a modified CRISPR strategy was utilized. A sgRNA was designed covering the sequence to be mutated (Figure 2A) and a pX330 plasmid constructed and validated *in vitro* [13]. Following validation the sgRNA was coinjected along with hCas9 mRNA and a single stranded oligonucleotide containing the S124A and S127A mutations as follows; oligonucleotide—100 ng/µL, hCas9/sgRNA—20/20 ng/µL, the number of fertilized eggs injected—115, the number of injected eggs transplanted into oviducts—104, the number of pups born—20, the number of gene modified (GM) pups—7 (Figure 2A). Of the GM pups obtained for *Dnaic1*, 5/7 (71.4%) had either S124A and S127A mutations or the S127A mutation only. Of the five pups with the targeted mutations 4/5 (80%) had both the S124A and S127A (Figure 2B) and 1/5 (20%) had only the S127A mutation. All four pups containing S124A and S127A mutations, designated *Dnaic1^em1Osb^* mice, also had other insertions or deletions (indels) in the opposite allele due to NHEJ that is caused by the CRISPR/Cas9 system.

#### 2.2.2. Fertility Analysis of *Dnaic1* Mutant Mice

Following selective breeding of the *Dnaic1^em1Osb/em1Osb^* mice, no overt abnormalities were observed in the body size, development or behavior of mice homozygous for this double amino acid substitution. To assess fertility of *Dnaic1^em1Osb/em1Osb^* male mice, natural mating analysis was performed. Control or homozygote males were caged with two wild-type (WT) females for approximately 3 months. Plugs were checked daily to determine copulation had occurred and litter size was examined. There was no significant difference in litter size between the control and *Dnaic1^em1Osb/em1Osb^* males (control; 9 ± 2.4, *Dnaic1^em1Osb/em1Osb^*; 8.5 ± 2.0, Figure 2C).

#### 2.2.3. Sperm Motility Analysis of *Dnaic1* Mutant Mice

Sperm motility analysis was performed using CASA (computer assisted sperm analysis) following incubation in TYH *in vitro* fertilization media (37 °C/5% CO_2_). There was no significant difference between the motility of the *Dnaic1^em1Osb/em1Osb^* samples when compared to controls in motility parameters (total motility, progressive motility, average path velocity; VAP, straight line velocity; VSL, curvilinear velocity; VCL) at either 10 or 120 min (Table S2).

### 2.3. Analysis of Wdr63 sKO Phenotype

#### 2.3.1. Generation of *Wdr63* KO Mice

**Figure 3:**
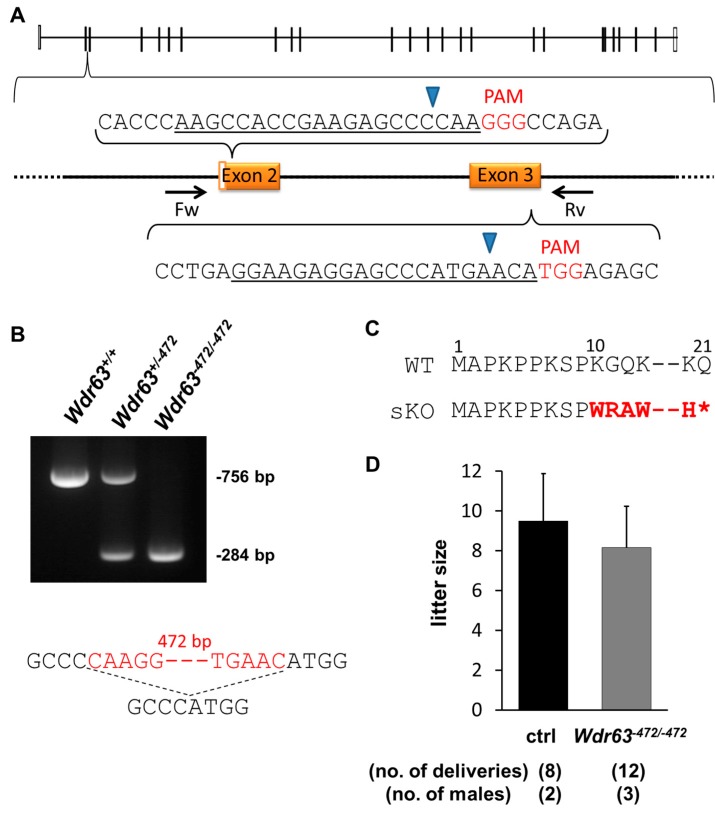
CRISPR/Cas9 strategy and results for *Wdr63^−472/−472^* male mice. (**A**) *Wdr63* exons 2 and 3 were targeted; coding exons shown as solid black bars, non-coding exons shown as open bars. Forward (Fw) and reverse (Rv) primers for validation and genotyping shown; black arrows labelled Fw and Rv. sgRNA sequence underlined in black. PAM sequence is shown in red. Blue arrowhead indicates site of Cas9 digestion; (**B**) PCR of WT, heterozygote (*Wdr63^+/−472^*) and homozygote (*Wdr63^−472/−472^*) mice; Sequence showing part of deleted region; deleted region in red (472 bp in length); dashed line indicates deleted region; (**C**) Frameshift mutation of *Wdr63^−472/−472^* mice, mutation shown in red, * indicates a premature stop codon. Amino acid number shown above sequence; (**D**) Natural mating analysis of *Wdr63^−472/−472^* male mice with WT females.

As *Wdr63* showed expression in different tissues (Figure 1A) a conditional KO (cKO) would be recommended. However, as the CRISPR/Cas9 system is fast and efficient it was simple to generate mice without a conditional KO strategy to assess whether these genes were essential for other body systems. For the KO of *Wdr63* two separate pX330 plasmids each containing a single sgRNA were constructed, approximately 500 bp apart and spanning the coding regions of exon 2 and 3 (Figure 3A). These were injected as follows; sgRNA 1/pX330 plasmid—5 ng/µL; sgRNA 2/pX330 plasmid 5 ng/µL; the number of fertilized eggs injected—119; the number of injected eggs transplanted into oviduct—80; the number of pups born—6; the number of GM pups—3. Of the six pups obtained 50% of them were GM. One hundred percent of GM pups had deletions of 472 bp (one pup) and 483 bp (two pups) due to targeted cutting by both sgRNAs, however these deletions occurred on only one allele in all three mice. Both pups with a deletion of 483 bp also had a deletion of 9 bp on the opposite allele, due to cutting by a single sgRNA. Selective breeding produced homozygous mice with a deletion of 472 bp.

Genomic PCR of *Wdr63* produces a product of 756 bp, whilst the KO mice contain a product of only 284 bp (Figure 3B), allowing for ease of genotyping of subsequent generations. Of the 472 bp deleted, 73 nucleotides encoded exons. This deletion resulted in a frameshift mutation (K10W) (Figure 3C) and the appearance of a stop codon 12 amino acids later causing a truncation of the WDR63 protein. We confirmed this premature stop codon via RT-PCR of *Wdr63^−472/−472^* testes (Figure S1A). Although two bands were detected (Figure S1A) sequencing showed both bands contained the mutation and the second (lower) band represented a splice variant. The splice variant was missing exon 4; however this exon comes after the mutation, which introduced a premature stop codon, indicating no protein was made after this point.

#### 2.3.2. Fertility Analysis of *Wdr63* KO Mice

No overt abnormality was observed in body size, development or behavior in *Wdr63^−472/−472^* mice. Fertility of *Wdr63^−472/−472^* male mice was determined by mating with two WT females for approximately three months. There was no significant difference in litter size between the control and *Wdr63^−472/−472^* males (control; 9.5 ± 2.4, *Wdr63^−472/−472^*; 8.2 ± 2.1, Figure 3D).

#### 2.3.3. Sperm Motility of *Wdr63* KO Mice

Sperm motility analysis was performed as above. There was no significant difference between the motility of the *Wdr63^−472/−472^* samples when compared to controls in motility parameters (total motility, progressive motility, VAP, VSL, and VCL) at either 10 or 120 min (Table S3).

#### 2.3.4. Expression Pattern of *Wdr78*

*Wdr78* is another component of the intermediate chain of the IAD (Table S1). To assess the possibility of compensation in the *Wdr63^−472/−472^* mice by *Wdr78* we performed RT-PCR tissue and testes age expression analysis. *Wdr78* is strongly expressed in the testes, as well as the thymus and weakly expressed in the brain (Figure S1B). In the testes, *Wdr78* is expressed weakly in weeks one and two, but expression increases in week three (Figure S1C), one week before the expression of *Wdr63* (Figure 1B). In the *Wdr63^−472/−472^* testes *Wdr78* was expressed to a similar level as the control (Figure S1D).

### 2.4. Analysis of Ccdc63 sKO Phenotype

#### 2.4.1. Generation of *Ccdc63* KO Mice

**Figure 4:**
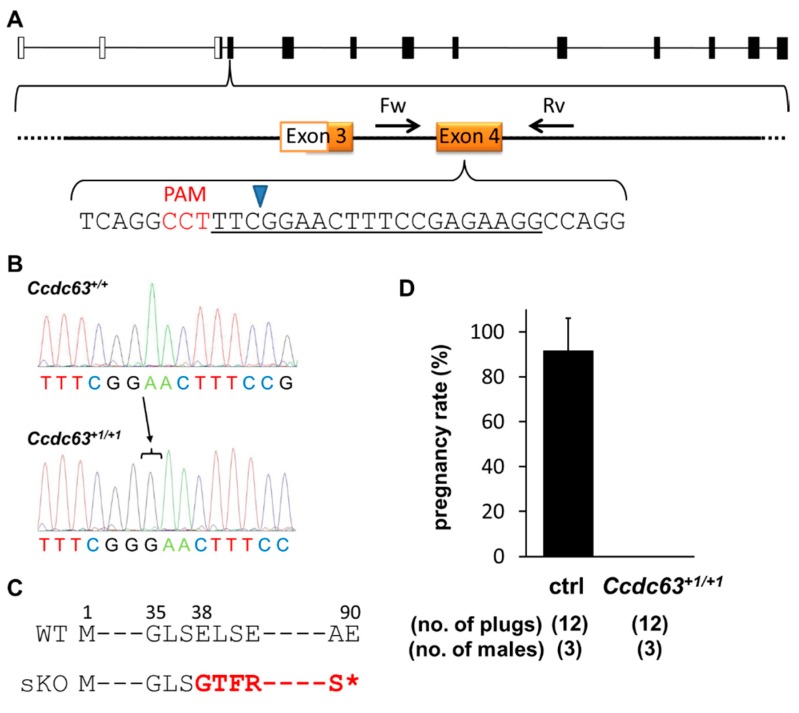
CRISPR/Cas9 strategy and results for *Ccdc63^+1/+1^* male mice. (**A**) *Ccdc63* exon 4 was targeted; coding exons shown as solid black bars, non-coding exons shown as open bars. Forward (Fw) and reverse (Rv) primers for validation and genotyping shown; black arrows labelled Fw and Rv. sgRNA sequence underlined in black. PAM sequence is shown in red. Blue arrowhead indicates site of Cas9 digestion; (**B**) Sequencing of *Ccdc63^+1/+1^* mice; black arrow and bar indicates the nucleotide inserted; (**C**) Frameshift mutation of *Ccdc63^+1/+1^*, mutation shown in red, * indicates a premature stop codon. Amino acid number shown above sequence; (**D**) Natural mating analysis of *Ccdc63^+1/+1^* male mice with WT females. Pregnancy rate (pregnancy/vaginal plug) presented as a percentage.

A simple knockout mouse of *Ccdc63* was made using the CRISPR/Cas9 system despite the fact that the gene is expressed in various tissues (Figure 1A). However, unlike *Wdr63* where two separate plasmids were constructed, in this case only a single sgRNA-containing pX330 plasmid was injected into fertilized oocytes. The target region was the coding sequence of exon 4 (Figure 4A). The plasmid was injected as follows; sgRNA/pX330 plasmid—5 ng/µL, the number of fertilized eggs injected—112, the number of injected eggs transplanted into oviducts—87, the number of pups born—21, the number of GM pups—7. Of the seven GM pups, 2/7 (28.6%) were mosaic, 1/7 (14.3%) was heterozygote, 1/7 (14.3%) was homozygote and 3/7 (42.9%) had insertions or deletions (indels) on both alleles. Mosaicism was detected through sequencing by the expression of three waves (Figure S2A). The homozygote pup had a deletion of 9 bp on both alleles, potentially causing an in-frame mutation and was therefore not selected for breeding. The heterozygote pup also had a deletion of 9 bp and was also not selected. Breeding of the mosaic pups generated mice with a WT, 1 bp insertion, and 18 bp (GCTCTTCAGGCCTTTCGG) deletion, confirming that the F0 mouse was mosaic. The mouse line with 1 bp insertion (Figure 4B) was chosen for breeding and analysis. This insertion resulted in a frameshift mutation of E38G (Figure 4C) with a premature stop codon introduced 53 amino acids later. This stop codon was confirmed via RT-PCR of *Ccdc^+1/+1^* testes. Even though two bands were detected (Figure S2B) sequencing showed both bands contained the mutation and the second (lower) band represented a splice variant. The splice variant was missing the first 63 bp of exon 4, and this sequence comes before the mutation; however 1 bp insertion in the later part of exon 4 introduced the same premature stop codon, therefore neither variants produced the full length proteins.

#### 2.4.2. Fertility Analysis of *Ccdc63* KO Mice

No overt abnormality was observed in body size, development or behavior of *Ccdc63^+1/+1^* mice. Male *Ccdc63^+1/+1^* mice and controls were caged with two WT females for approximately three months. Over this time period there were no litters born, despite regular copulation (Figure 4D). To ensure this phenotype arose from the sKO of *Ccdc63*, off-target analysis for this gene was performed on the F0 mice. No mutations were found amongst three off-target candidates using a 12 bp matching sequence plus PAM sequence search (Table S4).

#### 2.4.3. Morphological Analysis of *Ccdc63* KO Testes and Spermatozoa

**Figure 5:**
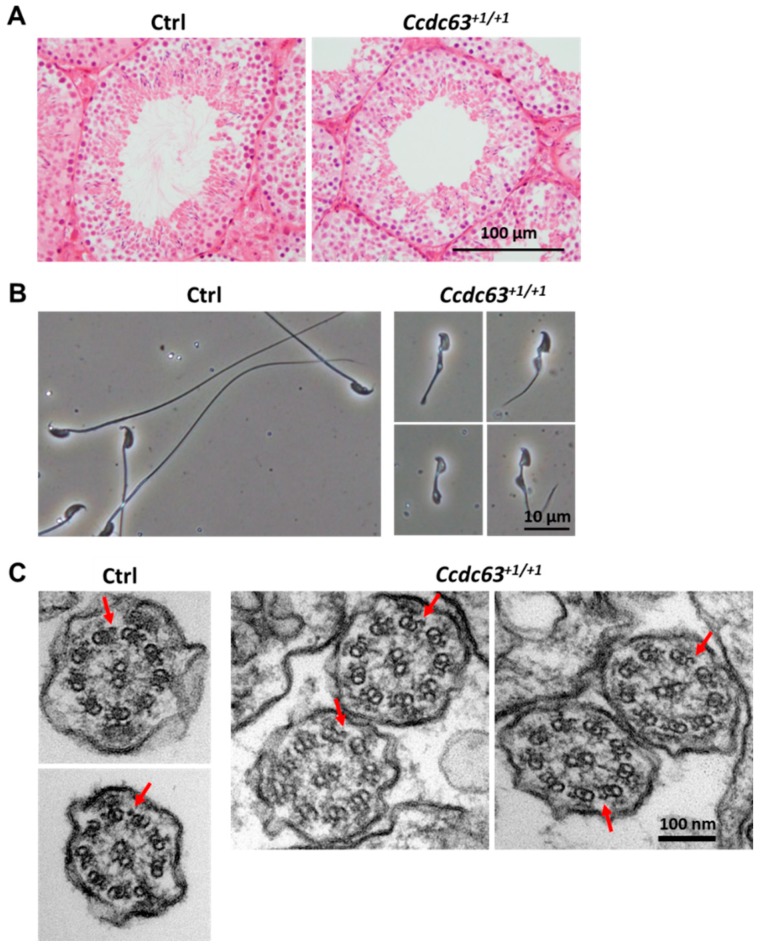
Histological and morphological analysis of *Ccdc63^+1/+1^* mice testis and spermatozoa; (**A**) Testis section of control (Ctrl) and *Ccdc63^+1/+1^* mice stained with Hematoxylin/Eosin (**B**) Sperm morphology of control and *Ccdc63^+1/+1^* mice; (**C**) Electron micrograph of a cross section of the end piece of sperm flagella of control and the flagella of *Ccdc63^+1/+1^* mice. Red arrowheads indicate OAD.

Morphological analysis of testes from *Ccdc63^+1/+1^* males showed the presence of spermatids with a developing acrosome, but lacking elongating flagella (Figure 5A and Figure S3A,B). Further analysis of the spermatozoa collected from the cauda epididymis showed severe defects in the morphology of the sperm heads and flagella (Figure 5B). No motile spermatozoa were observed in *Ccdc63^+1/+1^* mice. These results indicate that *Ccdc63* is necessary for spermiogenesis, especially for the elongation of the flagellum.

To examine if KO spermatozoa can activate oocytes, we performed intracytoplasmic sperm injection (ICSI) using testicular spermatozoa. The *Ccdc63* null spermatozoa-injected oocytes developed to 2 cell stage embryos at a comparable rate to those injected with control spermatozoa (control 46.9%, 2 cell/injected oocytes = 15/32; *Ccdc63^+1/+1^* 48.1%, 2 cell/injected oocytes = 26/54). Transferred 2 cell embryos developed to term (control 33.3%, pups/transfer = 2/6; *Ccdc63^+1/+1^* 27.8%, pups/transfer = 5/18).

As *Ccdc63* is the homologue of *Chlamyomonas* DC2 that is part of the dynein docking complex and is found on the OAD it was hypothesized that there would be a further defect in this structure. Electron microscopy showed, however, that the 9 + 2 structure of the axoneme including OAD was intact in the spermatozoa of *Ccdc63^+1/+1^* mice (Figure 5C). It should be noted that following the electron microscopy no accessory structures were found suggesting there may also be a problem in correct formation of the outer dense fibers, the mitochondrial sheath and the fibrous sheath (Figure 5C). This phenotype can also be seen under phase contrast microscopy (Figure 5B).

As *Ccdc63* is the testis specific homologue of *Ccdc114* it was thought that there may be some compensation, allowing the OAD to remain unaffected by the loss of *Ccdc63*. To investigate if this was likely, the location and expression of *Ccdc114* was checked by RT-PCR (Figure S4A,B). *Ccdc114* was expressed also in the testes, appearing from one week of age, but with increased expression at three weeks, two weeks before *Ccdc63* appears (Figure 1B and Figure S4B). *Ccdc114* is expressed normally in the *Ccdc63^+1/+1^* testes (Figure S4C).

### 2.5. Discussion

In this study, we have generated three mouse lines deleting or mutating genes related to the dynein complex using several variations of the CRISPR/Cas9 system. We successfully generated a double amino acid substitution in *Dnaic1*, resulting in serine residues 124 and 127 becoming alanine residues. We also deleted a 472 bp region of *Wdr63*, including coding sequence across two exons and we produced an insertion in *Ccdc63*, both resulting in a frameshift mutation and a premature stop codon.

The use of CRISPR/Cas9 allowed all of these gene-manipulated animals to be produced in about two months. The generation of the amino acid substitutions in *Dnaic1* was relatively efficient, with approximately 71% of GM mice containing one or both of the desired mutations. This high efficiency could be due to the fact that the sgRNA target site covered the site of mutation, as it has been shown that the closer the point of sgRNA digestion is to the desired mutation locus, the more efficiently the desired mutation occurs [27,28]. Our data further supports this as the mutation closer to the site of digestion was achieved in more GM pups than the further mutation (5/7 pups had S127A *vs*. 4/7 with S124A; site of digestion 10 bp downstream from S124, but only 1 bp downstream from S127; Figure 2A). It is also possible a sKO of *Dnaic1* is embryonically lethal [16,17], which could account for the higher ratio of desired mutation seen in this study.

Upon analysis we demonstrated that the *Dnaic1^em1Osb/em1Osb^* male mice were fully fertile, with no apparent difference in their sperm motility (Table S2). We can conclude from this that the phosphorylation of these two serines (124 and 127) is not essential to flagella formation, acquisition of motility, or to the fertilizing ability of the spermatozoa, in mice.

Another use of the CRISPR/Cas9 system is to delete larger regions of a gene by co-injection of two sgRNAs. With the sKO of *Wdr63* we have shown that this is also efficient, with 100% of GM pups containing deletions spanning the two target regions. However, *Wdr63* sKO males showed no impairment in fertility. As *Wdr63* is a component of the IAD and was highly conserved, it was thought that it would be involved in bend formation or motility of the mouse spermatozoa. This was not seen, suggesting that other intermediate chains of the IAD such as *Wdr78* or *Casc1* may compensate for this role in mouse sperm motility. In *Chlamydomonas*, however, the mutant of *IC140* (also known as *Ida7*, homolog of *Wdr63*) lacks IAD (f/l1) and shows a motility defect [29], suggesting that there is no compensation by *IC138* in *Chlamydomonas* (*Chlamydomonas* homolog of *Wdr78*). Further research is necessary to examine if there is compensation in mice, by mutating *Wdr78* or both *Wdr63* and *Wdr78*. It should be noted that it is still unclear what the components of the IAD are in mouse spermatozoa.

The final use of CRISPR/Cas9 was the injection of the single sgRNA to generate a sKO of *Ccdc63*. This variant of the CRISPR/Cas9 plasmid injection resulted in heterozygote, homozygote and mosaic GM mice. The male *Ccdc63^+1/+1^* mice obtained after breeding were infertile, with shortened sperm tails and malformed sperm heads. However, these spermatozoa were able to activate oocytes when used in ICSI and these embryos were able to develop to term. This indicates that the *Ccdc63* null spermatozoa’s genomic integrity is intact.

The hypothesis was that *Ccdc63* would be important for the formation of the OAD, as the amino acid sequence is similar to that of *Chlamydomonas* DC2 that is a component of the ODA-DC. Yet EM analysis of the sKO spermatozoa showed no obvious defect in the OAD of the spermatozoa from these mice. This could be simply due to compensation by *Ccdc114*, the somatically expressed homologue of *Ccdc63* known to be involved in PCD, as it is also present in the testis; in fact it appears in the testis approximately two weeks before *Ccdc63* (Figure 1B and Figure S4B). This could explain the correct assembly of the OAD. Our results indicate that *Ccdc63* individually plays a critical role in the formation of sperm heads and flagella during spermiogenesis but not OAD assembly. It would be interesting to mutate *Ccdc114* or both *Ccdc114* and *Ccdc63* to assess their individual and combined functions in the OAD assembly in spermatozoa.

With the ease and efficiency of the CRISPR/Cas9 system we have demonstrated how it can be applied to investigate a complex group of related genes, in this case the dyneins. Whilst *Dnaic1^em1Osb/em1Osb^* mice showed no fertility phenotypes, we demonstrated the ease of amino acid substitution using the CRISPR/Cas9 system via targeted mutation with HDR. It is interesting to note that other KO mice in the dynein complex also exhibit failure in flagellar elongation [30,31,32,33], suggesting that there is a quality control mechanism that triggers abortion of spermiogenesis when the flagellar structural proteins are defective [30]. If this is the case, further research whereby specific amino acids are mutated without changing the overall configuration of the structural proteins, (such as the dyneins), as it was done here with *Dnaic1*, bypassing this quality control mechanism would allow the analysis of the functions of specific domains in regulating flagellar motility. As several cases of male infertility in humans are due to point mutations or similarly small mutations [34,35,36,37], the ability of the CRISPR/Cas9 system to manipulate the genome in this subtle way opens up new avenues to understanding the complexity and machinery of male factor fertility.

## 3. Experimental Section

### 3.1. Animals

All animal experiments were approved by the Animal Care and Use committee of the Research Institute for Microbial Diseases, Osaka University (Osaka, Japan).

### 3.2. RT-PCR to Determine Gene Expression

RT-PCR was performed as described previously [38]. In brief, RT-PCR was done using 10 ng of cDNA from various tissues from C57BL/6 mice using the forward and reverse primers as follows; *Dnaic1* 5′-CGAACTTTTCAGCCACAGCC-3′ and 5′-CCGTGTCCCACTGCAAAAAG-3′; *Wdr63* 5′-TGACCCCAATATCATCGCCG-3′ and 5′-CTGGGAGTTTGCGTTGTTGG-3′; *Ccdc63* 5′-CACGTCTACCAGCAGCTTCA-3′ and 5′-ATCCGCTGGGTCTTCTTGTG-3′; *Ccdc114* 5′-AGGTGCAGGTGGAAATCGAG-3′ and 5′-TCCGGTTGTCGTTCATCGTT-3′; *Wdr78* 5′-GGCCTTGATATCCCGACAGG-3′ and 5′-TGGGCCCAAGCAGAATGTTG-3′; β*-actin* 5′-AAGTGTGACGTTGACATCCG-3′ and 5′-GATCCACATCTGCTGGAAGG-3′, respectively.

RT-PCR was also performed using 10 ng of testicular cDNA from 1–5 weeks old C57BL/6 male mice using the same primers as above.

RT-PCR was also undertaken using RNA from the *Wdr63^−472/−472^* and *Ccdc63^+1/+1^* testes. The primers used were as follows; *Wdr63* 5′-AGAAGAGTCAGACAGACTCCC-3′ and 5′-GGAGGATTGTAGACGTACACG-3′; *Ccdc63* 5′-CTCAGGCGAGGGGACAATAC-3′ and 5′-AGAGCTGTCTCTGGTTCGTG-3′. The primers for *Wdr78* and *Ccdc114* were the same as the primers mentioned above.

The amplification conditions were 30 s at 94 °C, followed by 35 cycles for the experiments shown in Figure 1A, 30 cycles for data in Figure 1B, Figures S1B,C and S4A,B, and 40 cycles for the experiments shown in Figures S1A and S2B. Semi-quantitative analysis shown in Figures S1D and S4C were done at 10, 20, 30 and 40 cycles. Cycles were 94 °C for 30 s, 60 °C for 30 s (65 °C for 30 s for Figure S1A) and 72 °C for 30 s (68 °C for 30 s for Figures S1A,D, S4C and 68 °C for 1 min for Figure S2B), with a final 2 min extension at 72 °C using KOD Neo FX Taq polymerase (Toyobo Life Sciences, Osaka, Japan) or at 68 °C using Platinum Taq DNA polymerase (ThermoFisher Scientific, Waltham, MA, USA) (Figures S1A,D, S2B and S4C).

### 3.3. Plasmid and Oligonucleotide Preparation

Potential off-target sites were found using free soft-ware, Bowtie [39] with rules outlined previously [40,41,42]. Twelve bases preceding the PAM sequence with AGG, GGG, CGG, and TGG were aligned with the mouse genome (mm9). The sgRNAs with the minimum of off-target sites were chosen for validation.

For the simple knockouts of *Wdr63* and *Ccdc63* and the point mutation of *Dnaic1* the plasmids expressing hCas9 and sgRNA were prepared by ligating oligos into the BbsI site of pX330 (http://www.addgene.org/42230/; [43]). The sgRNA targets were as follows; *Wdr63* gAAGCCACCGAAGAGCCCCAA and GGAAGAGGAGCCCATGAACA; *Ccdc63* gCCTTCTCGGAAAGTTCCGAA (N.B. the initial “g” was added as at the time of generation this was thought to be necessary for sgRNA expression driven by U6 promoter). The pCAG-EGxxFP target plasmid was prepared as previously [13]. Validation of sgRNA/CRISPR cleavage activity was done by transfection of HEK293T cells as previously reported [13].

Following validation of the sgRNAs for *Dnaic1*, the sgRNA target was as follows; *Dnaic1* gAGGTTCACAGGAGTCTATCA (N.B. the initial “g” was added as at the time of generation this was thought to be necessary for sgRNA expression driven by U6 promoter) and mRNA for the hCas9 as shown previously [13]. A single stranded oligonucleotide containing the desired mutation with approximately 60nts homologous to the target region on either side was designed and ordered. The oligonucleotide sequence was as follows; 5′-AGAGCCCAAGGGACAGAGTCTGAAAGACGGGAAGACGGTGACTGAGCTGGCTTGGACCCTTAACTCATCAACAATATCCTGTCAAGGTgccCAGGAGgccATCAAGGTGGTGACTTCAGAAGCAGAAAAC-3′.

### 3.4. Pronuclear Injection

B6D2F1 female mice were superovulated and mated with B6D2F1 males, and fertilized eggs were collected from the oviduct. The pX330 plasmids, sgRNA, Cas9 mRNA and/or oligonucleotides were injected into one of the pronuclei. The injected eggs were cultivated in potassium simplex optimization medium (kSOM) [44] overnight then two-cell stage embryos were transferred into the oviducts of pseudopregnant ICR females. The pups were genotyped by PCR and subsequent sequence analysis.

### 3.5. Genotyping

PCR of genomic DNA was done for *Dnaic1*, *Wdr63* and *Ccdc63* using the following primer sets; *Dnaic1* forward (plus EcoRI restriction enzyme sequence) 5′-aagaattcCAAAGACTCAGATGAAGGCCGT-3′, reverse (plus BamHI restriction enzyme sequence) 5′-aaggatccCCCTGACCTGGTGAAATGGGTA-3′; *Wdr63* forward (plus EcoRI restriction enzyme sequence) 5′-gggaattcGATCTAGTCCAGGCCCAAACC-3′ and reverse (plus BamHI restriction enzyme sequence) 5′-ggggatccGATGCGATCTAAACCCTCGGG-3′; *Ccdc63* forward (plus EcoRI restriction enzyme sequence) 5′-gggaattcCTACCCTACGACAGACAGGC-3′ and reverse (plus BamHI restriction enzyme sequence) 5′-ggggatccGCTCACTGGATATCTTCCCG-3′. Direct sequencing of PCR products was then performed for *Dnaic1* and *Ccdc63*. Restriction enzymes were added as these primers were also used for pCAG-EGXXFP plasmid construction.

Mutant mice were assigned labels and deposited into the Riken BioResource Center with the following stock numbers; RBRC09507B6D2-Dnaic1<em1Osb>/S124A S127A; RBRC09505B6D2-Wdr63<em1Osb>; RBRC09506B6D2-Ccdc63<em1Osb>, as were the plasmids used to create them; pX330-Dnaic1/sgRNA#7; pX330-Wdr63/sgRNA#1, pX330-Wdr63/sgRNA#5; pX330-Ccdc63/sgRNA#3.

### 3.6. Off-Target Analysis of Ccdc63

There were three potential off-target sites for *Ccdc63*. PCR and sequencing analysis were performed for each off-target site. The primer sets for the off-target analysis were as follows; OT-1 5′-CAGGATCACGACAGGAGAGC-3′ and 5′-AACCCATCATGCTAGACGCC-3′; OT-2 5′-CACAGCCACAAGCTCAACAC-3′ and 5′-TCATCCTTCTGCCATCTCTTGTC-3′; OT-3 5′-TTTCCTTCCATAAGCTTTCC-3′ and 5′-CCATGGACAAGTACCAGTCC-3′.

### 3.7. Male Fertility Test

Sexually matured *Dnaic1^em1Osb/em1Osb^*, *Wdr63^−472/−472^* or *Ccdc63^+1/+1^* male mice were caged with two month-old B6D2F1 female mice for three months. Copulation was confirmed by checking for vaginal plugs every morning and the number of pups was counted on the day of birth.

### 3.8. ICSI

ICSI was performed as previously reported [45]. In brief, mature oocytes were collected from superovulated B6D2F1 mice 13–15 h after hCG injection. After hyaluronidase treatment to remove the cumulus oocyte complex, oocytes were placed in fresh CZB medium at 37 °C under 5% CO_2_ in air until subjected to ICSI. Each sperm head was separated from the tail by applying a few piezo pulses, then injected into an oocyte using a piezo manipulator (PrimeTech, Ibaraki, Japan) [46]. The next day, two-cell embryos were counted and transferred to pseudopregnant females. Pups were genotyped at birth.

### 3.9. Sperm Motility Analysis

Frozen spermatozoa from *Dnaic1^em1Osb/em1Osb^* and *Wdr63^−472/−472^* mice thawed and re-suspended in TYH medium [47]. Sperm motility was then measured using CEROS sperm analysis system (software version 12.3; Hamilton Thorne Biosciences, Beverly, MA, USA). Analysis settings described previously [48] was used.

### 3.10. Visualization of Ccdc63 Null Testes and Spermatozoa

Testes were collected from mature male mice and were fixed in 4% paraformaldehyde in PBS and processed for plastic embedding (Technovit 8100, Heraeus Kulzer, Wehrheim, Germany). The 5 µm plastic sections were stained with Periodic Acid Schiff (PAS) and then counterstained with Mayer’s hematoxylin solution (Wako, Osaka, Japan) or stained with hematoxylin and eosin (HE staining). The sections were then rinsed in 70% ethanol, dehydrated in increasing ethanol concentrations, and finally mounted in Entellan new (Merck, Osaka, Japan).

For electron microscopic observation, testicular samples were prepared for electron microscopy as previously described [49], and then examined using a JEM-1011 electron microscope (JEOL, Tokyo, Japan) at 80 kV.

### 3.11. Statistical Analysis

Statistical analyses were performed using student’s *t*-test. Differences were considered significant at *p* < 0.05 (*). Error bars shown as standard deviation (SD).

## 4. Conclusions

Here we have demonstrated the effective use of the CRISPR/Cas9 system to generate mutations to specifically targeted amino acids, as well as introducing premature stop codons in the mouse genome. We have shown that the CRISPR/Cas9 system can be quickly and easily used to generate mouse models for analyzing the complex components of the male reproductive system. One of these components, *Ccdc63*, was shown to not be involved in the formation of the outer dynein arms, but to be essential for spermiogenesis, adding to the body of knowledge about this family of proteins and their roles in male fertility.

## Supplementary Materials

Supplementary materials can be found at http://www.mdpi.com/1422-0067/16/10/24732/s1.
