# Supplementary material for: CRISPR/Cas9-Mediated Rapid Generation of Multiple Mouse Lines Identified Ccdc63 as Essential for Spermiogenesis
Source: Int J Mol Sci. 2015 Oct 16;16(10):24732–50. doi: 10.3390/ijms161024732 (PMC4632774; doi:10.3390/ijms161024732)
Supplement: Supplementary file 1 [file ijms-16-24732-s001.pdf]

Supplementary Information

Table S1. Conservation of dynein complex genes * between *Chlamydomonas reinhardtii* and *Mus musculus*.

***Chlamydomonas* Gene**	**Mouse (HUMAN) Gene**	**MGI /HGNC Number ****	**KO Mouse**
	Outer arm dynein (*OAD*)		
IC69	*Dnaic2 (DNAI2)*	2685574/18744	No
IC78	*Dnaic1 (DNAI1)*	1916172/2954	Yes (ciliary dyskinesia)
LC1	*Dnal1 (DNAL1)*	1921462/23247	No
LC2	*Tctex1d1 (TCTEX1D1)*	1914594/26882	No
	Tctex1d2 (TCTEX1D2)	1913311/28482	No
	Tctex1d4 (TCTEX1D4)	3045358/32315	No
	Tcte3 (TCTE3)	98642/11695	Yes (infertile; impaired spermatogenesis)
LC3/LC5	*Nme8 (NME8)*	1920662/16473	Yes (fertile)
	Nme9 (NME9)	4359686/21343	No
LC4	*No determined* (*ND*)	-	-
LC6/LC8/LC10	*Dnal4 (DNAL4)*	1859217/2955	No
	Dynll1 (DYNLL1)	1861457/15476	Yes (pre-weaning lethality)
	Dynll2 (DYNLL2)	1915347/24596	No
LC7	*Dynlrb1 (DYNLRB1)*	1914318/15468	No
	Dynlrb2 (DYNLRB2)	1922715/15467	No
DC1	*ND*	-	-
DC2	*Ccdc114 (CCDC114)*	2446120/26560	No
	Ccdc63 (CCDC63)	3607777/26669	No
DC3	*ND*	-	-
	Inner arm dynein (*IAD*) (*f/I1*)		
IC138	*Wdr78 (WDR78)*	2385328/ 26252	No
IC140	*Wdr63 (WDR63)*	3045269/30711	No
IC97	*Casc1 (CASC1)*	2444480/29599	Yes (higher incidence of lung tumour)
FAP120	*Ank2 (ANK2)*	88025/493	Yes (embryonically lethal)
TCTEX1	*Dynlt1a (DYNLT1)*	3807506/11697	No
	Dynlt1b (DYNLT1)	98643/11697	No
	Dynlt1c (DYNLT1)	3807476/11697	No
	Dynlt1f (DYNLT1)	3780996/11697	No
TCTEX2B	*Tctex1d1 (TCTEX1D1)*	1914594/26882	No
	Tctex1d2 (TCTEX1D2)	1913311/28482	No
	Tctex1d4 (TCTEX1D4)	3045358/32315	No
	Tcte3 (TCTE3)	98642/11695	Yes (infertile; impaired spermatogenesis)

* Note; only intermediate, light chains and docking complexes are listed. ** MGI URL: http://www.informatics.jax.org/; HGNC URL: http://www.genenames.org/.

Table S2. Mean computer assisted sperm analysis (CASA) values for *Dnaic1*$^{em1Osb/em1Osb}$ mice *vs.* control mice.

Mouse Genotype	Incubation Time (min)	Motility (%)	Progressive Motility (%)	Average Path Velocity (VAP) (μm/s)	Linear Velocity (VSL) (μm/s)	Curvilinear Velocity (VCL) (μm/s)
Ctrl	10	87.0 ± 7.9	67.7 ± 5.0	119.7 ± 6.7	90.1 ± 7.6	234.4 ± 5.4
	120	89.3 ± 4.0	57.0 ± 3.5	114.4 ± 9.4	75.5 ± 6.1	244.8 ± 34.7
Dnaic1$^{em1Osb/em1Osb}$	10	91.0 ± 6.1	73.3 ± 5.1	125.6 ± 7.3	96.2 ± 2.2	217.6 ± 29.5
	120	78.7 ± 17.0	54.0 ± 6.9	120.9 ± 4.7	73.3 ± 4.0	255.0 ± 8.8

Data are expressed as means ± SD (*n* = 3).

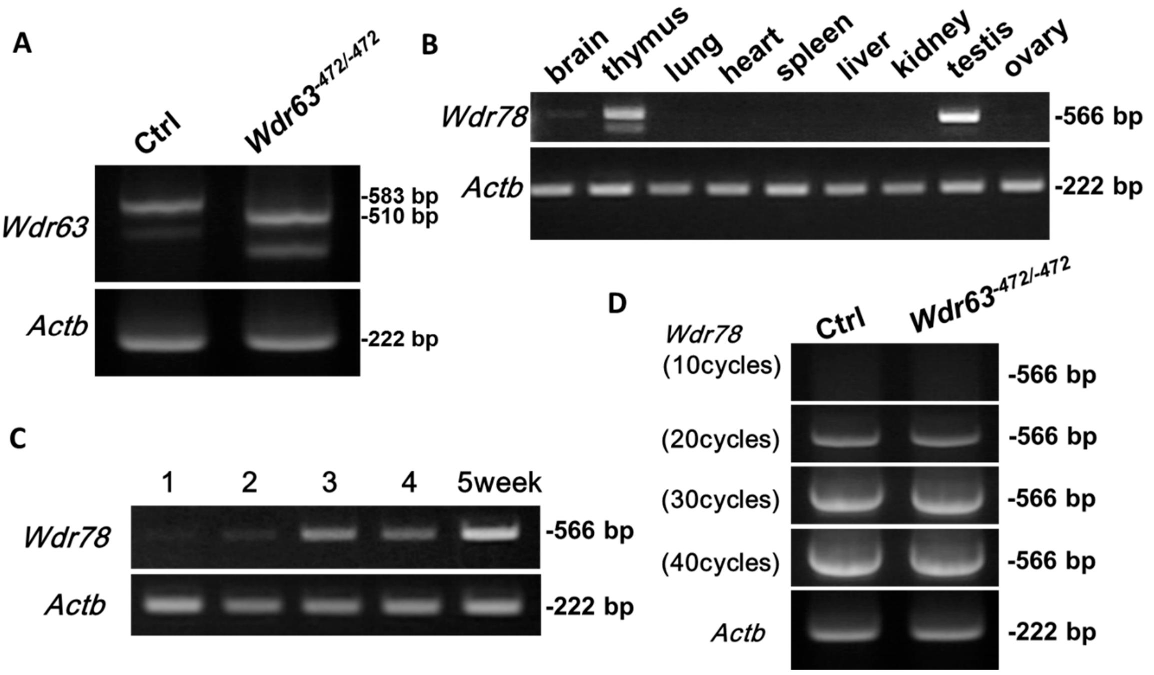

Figure S1. (**A**) RT-PCR for *Wdr63* in control and sKO testes; (**B**) RT-PCR for *Wdr78* in various tissues and (**C**) testes samples at different ages; (**D**) Semi-quantitative RT-PCR of *Wdr78* in control and sKO testes; *Actb* (β-*actin*) as control.

Table S3. Mean computer assisted sperm analysis (CASA) values for *Wdr63*$^{−472/−472}$ mice *vs.* control mice.

Mouse Genotype	Incubation Time (min)	Motility (%)	Progressive Motility (%)	Average Path Velocity (VAP) (μm/s)	Linear Velocity (VSL) (μm/s)	Curvilinear Velocity (VCL) (μm/s)
Ctrl	10	93.3 ± 4.7	75.0 ± 3.6	115.5 ± 4.6	87.4 ± 4.9	221.0 ± 21.6
	120	85.7 ± 13.6	54.3 ± 7.8	112.8 ± 14.2	71.1 ± 6.5	242.8 ± 37.4
Wdr63$^{-472/-472}$	10	93.3 ± 4.2	80.0 ± 7.5	117.3 ± 6.3	95.7 ± 6.2	188.0 ± 12.8
	120	91.0 ± 6.9	57.0 ± 5.0	103.3 ± 2.4	64.1 ± 4.4	188.7 ± 15.0

Data are expressed as means ± SD (*n* = 3).

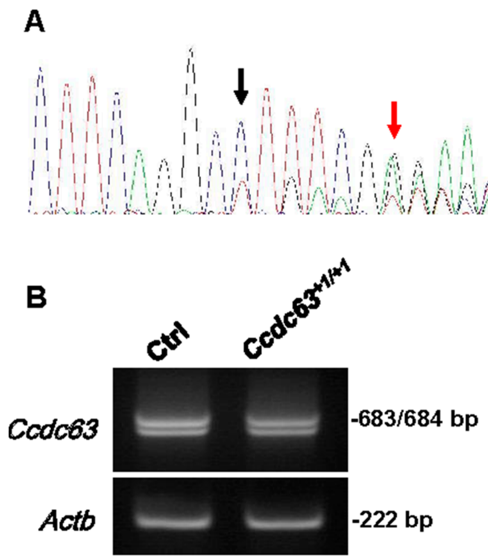

Figure S2. (**A**) Example of sequencing for a founder mosaic mouse. Black arrow indicates double wave (2 alleles) and red arrow indicates triple wave (3 alleles) (**B**) RT-PCR of *Ccdc63* in control and sKO testes. *Actb* (β-*actin*) as control.

Table S4. Off-target (OT) analysis of *Ccdc63* F0 mice.

Target	sgRNA Sequence + PAM*	Location	Mutation
Ccdc63	CCTTCTCGGAAAGTTCCGAAAGG	Chr5: 122129838-122129860	Yes (mosaic)
OT-1	AAAATGATGAAAGTTCCGAAGGG	Chr1: 132472528-132472550	No
OT-2	CCTTTCGGAACTTTCTAAATTGA	Chr4: 101217425-101217447	No
OT-3	CAGACTCTGAAAGTTCCGAAGGG	Chr19: 18320482-18320504	No

* PAM—protospacer adjacent motif; shown in red; 12 nucleotides homologous to *Ccdc63* target sgRNA underlined in black.

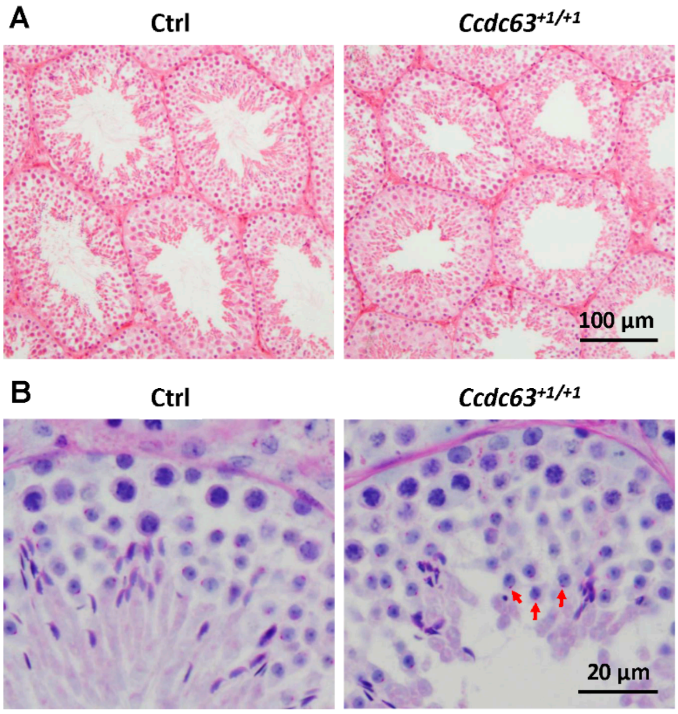

Figure S3. Morphological analysis of testes from control and *Ccdc63*$^{+1/+1}$ mice. (**A**) Hematoxylin/Eosin stained seminiferous tubules; (**B**) PAS/Hematoxylin staining of seminiferous tubules (stage III–IV), red arrows indicate developing acrosomes.

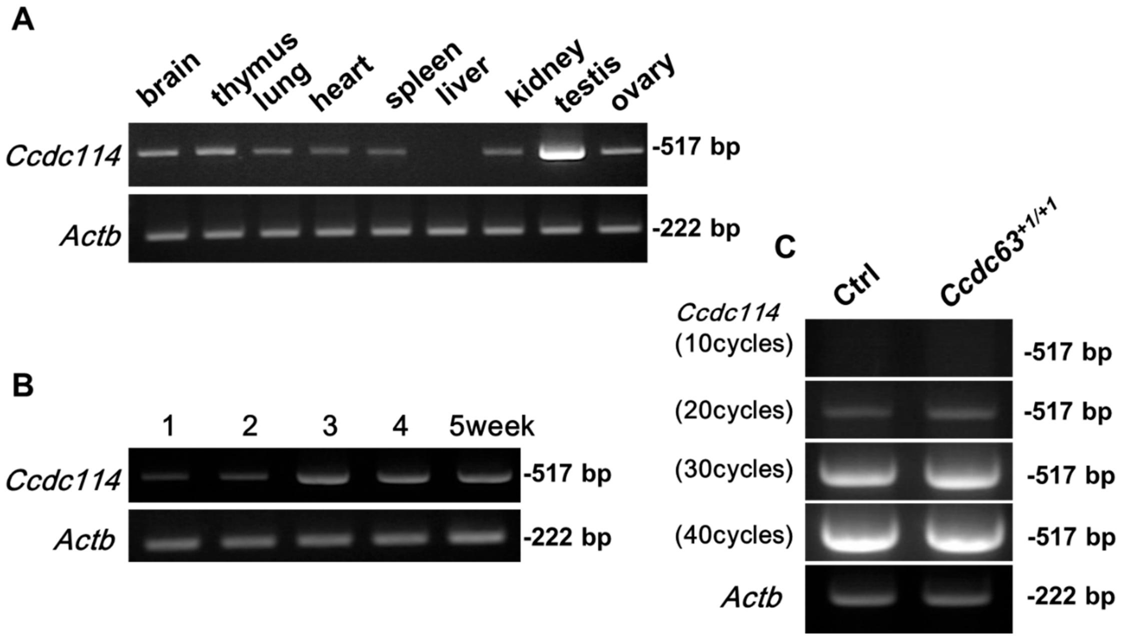

Figure S4. (**A**) RT-PCR for *Ccdc114* in various tissues and (**B**) testes samples at different ages. (**C**) Semi-quantitative RT-PCR of *Ccdc114* in control and sKO testes; *Actb* (β-*actin*) as control.
